# Relevance of MIC-1 in the Era of PSA as a Serum Based Predictor of Prostate Cancer: A Critical Evaluation

**DOI:** 10.1038/s41598-017-17207-2

**Published:** 2017-12-04

**Authors:** Navneeta Bansal, Deepak Kumar, Ashish Gupta, Deepak Chandra, Satya Narain Sankhwar, Anil Mandhani

**Affiliations:** 1 Department of Urology, King George’s Medical University, Lucknow, India; 2 Centre of Biomedical Research, SGPGIMS Campus, Lucknow, India; 3 Department of Biochemistry, Lucknow University, Lucknow, India; 4 Department of Urology, Sanjay Gandhi Post Graduate Institute of Medical Sciences, Lucknow, India

## Abstract

To reduce the ambiguity of contradictory observations in different studies regarding the expression level of Macrophage Inhibitory Cytokine-1 (MIC-1) in serum in prostate cancer (PC), benign prostatic hyperplasia (BPH) and healthy controls (HC), we designed this double-blind study. The study comprises 240 sera from PC, BPH and HC subjects. The expression level of MIC-1 in PC, BPH and HC were appraised using Western blot (WB) and ELISA based approach. WB and ELISA appraisal reveals that the expression level of MIC-1 is significantly higher in PC than in HC or BPH subjects. Regression analysis revealed a significant correlation between MIC-1 vs. PSA (r = 0.09; *p* < 0.001) and MIC-1 vs. GS (r = 0.7; *p* < 0.001). ROC analysis using discriminant predicted probability revealed that the MIC-1 was better than PSA. Moreover, the combination of MIC-1 and PSA was allowing 99.1% AUC for the differentiation of BPH + PC from HC, 97.9% AUC for differentiation of BPH from HC, 98.6% AUC for differentiation of PC from HC, and 96.7% AUC for the differentiation of PC from BPH. The augmented expression of MIC-1 in PC compared to BPH and HC subjects is in concurrent of the over-expression of MIC-1 in PC reports and confiscates the contradictory findings of other studies.

## Introduction

Despite much advancement in modern analytical techniques to identify cancer, prostate cancer (PC) still jeopardizes our community. PC is the sixth leading malignancy that engenders mortality among men worldwide^1^. Recent estimates of the PC burden project 161,360 new cases and 26,730 deaths during 2017 in US alone^2^. Conventional clinical prognosticators of PC severity and progression include - digital rectal examination (DRE), serum prostate-specific antigen (PSA), trans-rectal ultrasound (TRUS), and TRUS-guided histopathological staging (GS)^3,4^. PSA has been the linchpin of prostate cancer screening since the Food and Drug Administration (FDA) approved the PSA test in 1994. The application of PSA as a screening tool for PC has reduced death rates and the prevalence of advanced stage disease at diagnosis^5,6^. Though PSA provides a sensitive marker for PC diagnosis, it is not confined to PC only. Elevated serum PSA levels have been reported in benign prostatic hyperplasia (BPH) or prostatitis as well^7,8^. Thus there is a continual debate over the efficacy of PSA as a diagnostic marker (9). According to statistical data published by U.S. Preventive Services Task Force (USPSTF), 5.2 million U.S. dollars would be spent for PSA screening to prevent one death from PC^9^. These extraordinary costs, time and the overdiagnosis associated with PSA screening highlight the urgent need to determine a more efficient biomarker with improved specificity.

It is usually thought that clinically significant biomarkers may not correspond to the classical serum proteins but are rather instigated from secretion or shedding or leakage of proteins from specific tissue sites^10^ and thus provide the imperative biomarkers to reveal proteins expression changes present at different stages. The presence of highly abundant proteins such as albumin and immunoglobulins, however, might mask the lower abundance of proteins present in the serum; hence, depletion of serum prior to proteomic analysis has developed into a mainstay of clinical proteomic studies. New methods and protocols to decipher low-molecular-weight proteins (LMWP) for PC have been employed and executed to enrich and detect physiologically important LMWP^11,12^.

The Multiple Affinity Removal System (MARS-Hu6) column manufactured by Agilent Technologies^13,14^ removes the 6 most highly abundant proteins from serum namely albumin, IgG, anti-trypsin, IgA, transferrin and haptoglobin, and facilitates the appraisal of low abundant perturbed protein expression happening at different stages of PC development. Such a method would prospectively address the clinical dilemma within this disease: differentiating clinically indolent disease from aggressive forms of PC. The following serum proteins have been investigated for PC diagnosis and prognosis using highly advanced approaches: Afamin, alpha-2-HS-glycoprotein chain B, alpha-2-macroglobulin, zinc-α2-glycoprotein (ZAG), pentraxin 3 (PTX3), spondin 2 (Spon 2), follistatine (FST), pigment epithelium-derived factor (PEDF), fibronectin 1, chromogranin A, and alpha-1-antichymotrypsin^15^.

Macrophage Inhibitory Cytokine 1 (MIC-1) protein biomarkers have been investigated and perceived to be potential valuable biomarkers for PC identification and prognosis. MIC-1 or growth differentiation factor 15 (GDF-15) or non-steroidal anti-inflammatory drugs (NSAIDs) activated gene (NAG-1), is a member of the transforming growth factor beta superfamily. Studies have exhibited that MIC-1 expression is strongly associated with cancer development and progression^16,17^. Few studies have demonstrated elevated levels of MIC-1 in PC serum samples compared to normal samples^17,18^. Conversely, other studies have reported reduced expression of MIC-1 in PC serum samples^19,20^. These controversial results of different studies create ambiguity regarding the expression pattern of MIC-1 in respect to PC. To reduce this haze and learn more about the role of MIC-1 in PC, we designed this study with the following aim: to further evaluate MIC-1 protein biomarkers for clinical efficacy using the following approaches that may provide more unified vision for PC staging, grading, therapeutic monitoring (i) a double-blind study, (ii) appraisal of correlation of MIC-1 expression vs. PSA level, and (iii) appraisal of correlation among MIC-1 vs. GS.

## Results

The clinico-pathological analysis data of all participants in this study are presented in Table 1. Because albumin and other high-abundance proteins occur at ~80–90% in serum and usually create obstacles for accurate proteomic analysis, removing these high-abundance proteins from serum samples can augment the viability of low-abundance proteins and peptides, and facilitate precise analysis. In this study, high-abundance proteins were eliminated as the first step and WB following ELISA analysis was performed as the second step.

**Table 1:** Summary of clinical information’s of healthy control (HC), benign prostate hyperplasia (BPH) and prostate cancer (PC) patients.

Characteristics	HC	BPH	PC	Significance level
No. of subjects	80	75	85	
Age (mean ± SD)	61 ± 6	62 ± 7	63 ± 7	*p* = 0.08 (PC vs HC)
Low grade (LG)			63 ± 5	*p* = 0.77 (BPH vs HC)
High grade (HG)			63 ± 8	*p* = 0.16 (PC vs BPH)
				*p* = 0.91 (LG vs HG)
Gender				
Male	80	75	85	
Female	0	0	0	
Batch analysis				
HC (n = 80)	n = 35			*p* = 0.92 (for HC)
	n = 45			
BPH (n = 75)		n = 40		*p* = 0.16 (for BPH)
		n = 35		
PC (n = 85)				*p* = 0.88 & 0.86 (for PC)
LG (n = 40)			n = 25,15	
HG (n = 45)			n = 20,25	
BMI (mean ± SD)	22.8 ± 2.4	22.9 ± 3.1	23.1 ± 2.9	*p* = 0.55 (HC vs BPH)
Low grade (LG)			22.9 ± 3.2	*p* = 0.44 (HC vs PC)
High grade (HG)			23.3 ± 2.6	*p* = 0.92 (BPH vs PC)
Prostate Specific Antigen	2.0 ± 0.96	19.1 ± 27.9	32.5 ± 41.9	*p* = 0.001 (HC vs BPH)
Low grade (LG)			20.3 ± 30.9	*p* = 0.001 (HC vs PC)
High grade (HG)			41.5 ± 48.3	*p* = 0.02 (BPH vs PC)
				*p* = 0.03 (LG vs HG)
MIC-1	370 ± 107	621 ± 105	1536 ± 483	*p* = 0.001 (HC vs BPH)
				*p* = 0.001 (HC vs PC)
Low grade (LG)			1405 ± 486	*p* = 0.001 (BPH vs PC)
High grade (HG)			1653 ± 454	*p* = 0.017 (LG vs HG)
Digital Rectal Examination				
Grade 1,2,3	2.0 (1.0–2.0) ± 0.50	2.0 (1.0–3.0) ± 0.74	2.0 (1.0–3.0) ± 0.75	*p* = 0.04 (HC vs BPH)
				*p* = 0.001 (HC vs PC)
Low grade (LG)			Grade 1–3	*p* = 0.045 (BPH vs PC)
High grade (HG)			Grade 1–3	*p* = 0.040 (LG vs HG)
TRUS (symbolized as) 1,2,3	3.0 (2.0–3.0) ± 0.50	2.0 (1.0–3.0) ± 0.71	2.0 (1.0–2.0) ± 0.50	*p* = 0.7 (HC vs BPH)
				*p* = 0.001 (HC vs PC)
				*p* = 0.001 (BPH vs PC)
Gleason Score				
Low grade (LG)	0	0	6–7	
High grade (HG)	0	0	8–10	
Medications	0	0	0	

hypoechoic lesion denoted as 1, hyperechoic lesion denoted as 2, isoechoic lesion denoted as 3

PSA levels were appraised by one-way ANOVA followed by a post hoc Student-Newman-Keuls multiple comparisons test, categorical variables (DRE and TRUS) were assessed by nonparametric ANOVA followed by Kruskal-Wallis multiple comparisons test and paired t-test was applied between LG vs. HG cases as well as batch analysis. DRE and TRUS are presented as median (range) ± S.D.

**Figure 1:**
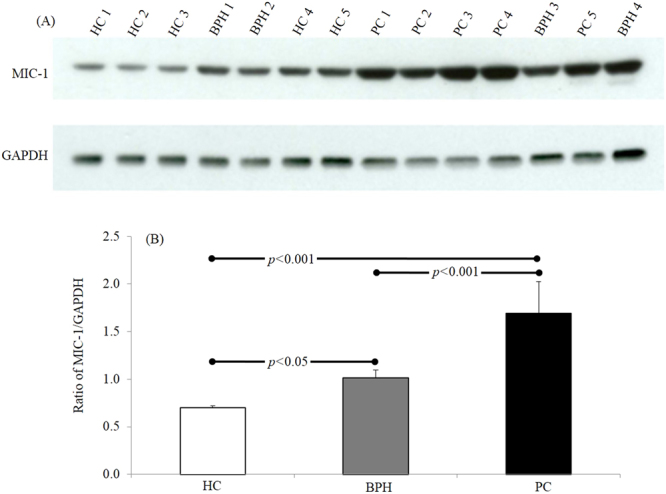
Expression pattern of MIC-1 protein through western blot analysis in double blinded serum samples obtained from HC (n = 5), BPH (n = 4), and PC (n = 5). (**A**) Western blots and (**B**) summary results. All 14 samples were executed simultaneously. MIC-1 and GAPDH clearly delineated with white space.

**Figure 2:**
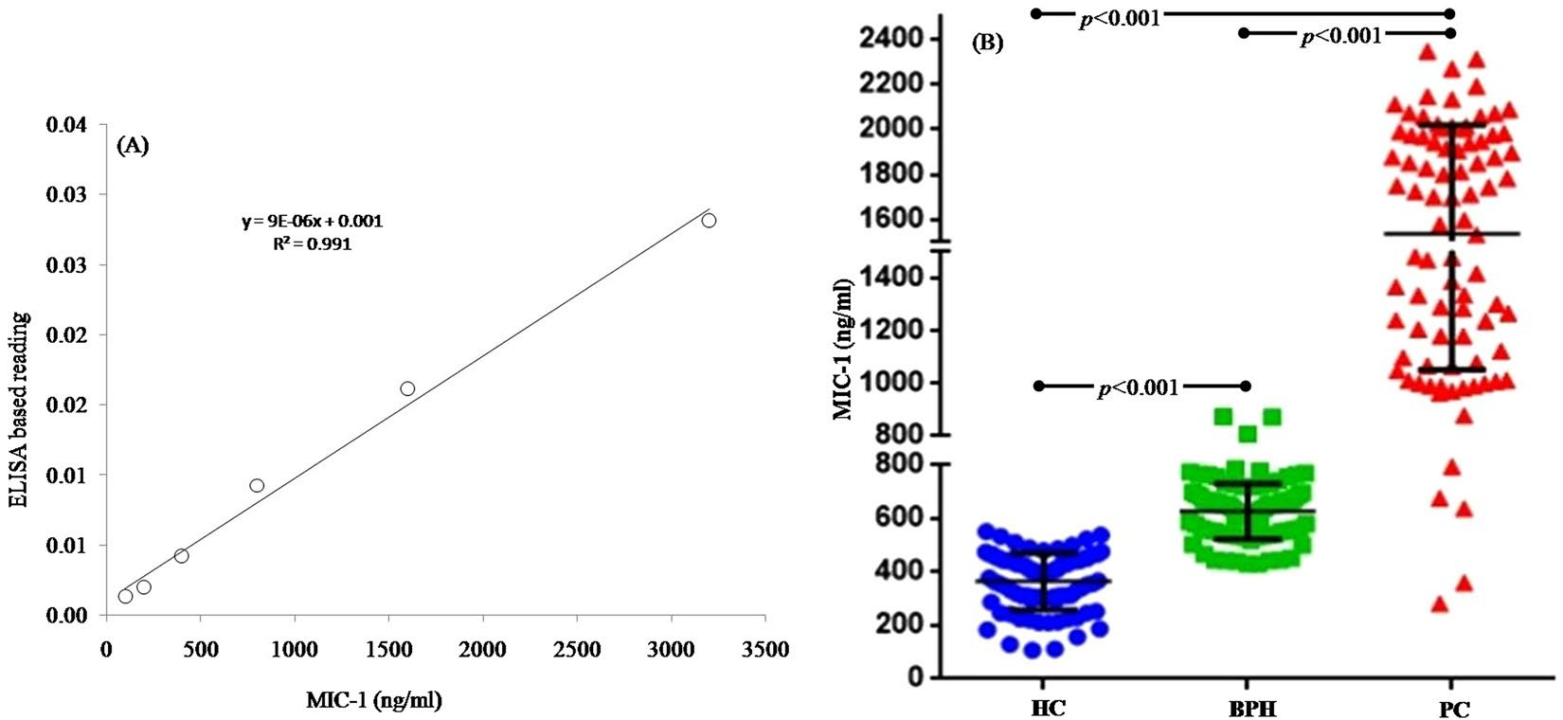
Measurement of absolute amount of MIC-1 in double blinded serum samples using calibration curve. (**A**) Calibration curve drawn with different concentration of standard MIC-1 proteins (**B**) dot plot of absolute amount of MIC-1 measured in double blinded serum samples obtained from HC (blue), BPH (green), and PC (red) using calibration curve equation.

**Figure 3:**
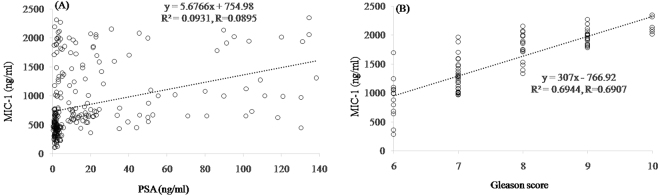
Regression analysis between (**A**) PSA versus MIC-1 and (**B**) MIC-1 verses Gleason score. The correlation is extremely significant (*p* < 0.001) between PSA versus MIC-1 and MIC-1 verses Gleason score.

The observation of WB analysis revealed that the expression level of MIC-1 was significantly higher in PC compared to HC and BPH (Fig. 1). Total 15 samples comprising HC (n = 5), BPH (n = 4), and PC (n = 5) were executed for WB. Figure 1A shows representative figure of WB and bar graph is the average of MIC-1/GAPDH ratio measured in each cohort (Fig. 1B). ELISA based analysis further confirmed the finding of WB observations (Fig. 2). Regression analysis of MIC-1 vs. PSA level revealed (r = 0.09) the significant correlation (p < 0.001). Similarly, regression analysis of MIC-1 vs. GS revealed (r = 0.7) the significant correlation (p < 0.001) (Fig. 3) (Supplementary data). Higher GS and advanced cancers showed considerably augmented levels of MIC-1 compared to those with low GS.

**Figure 4:**
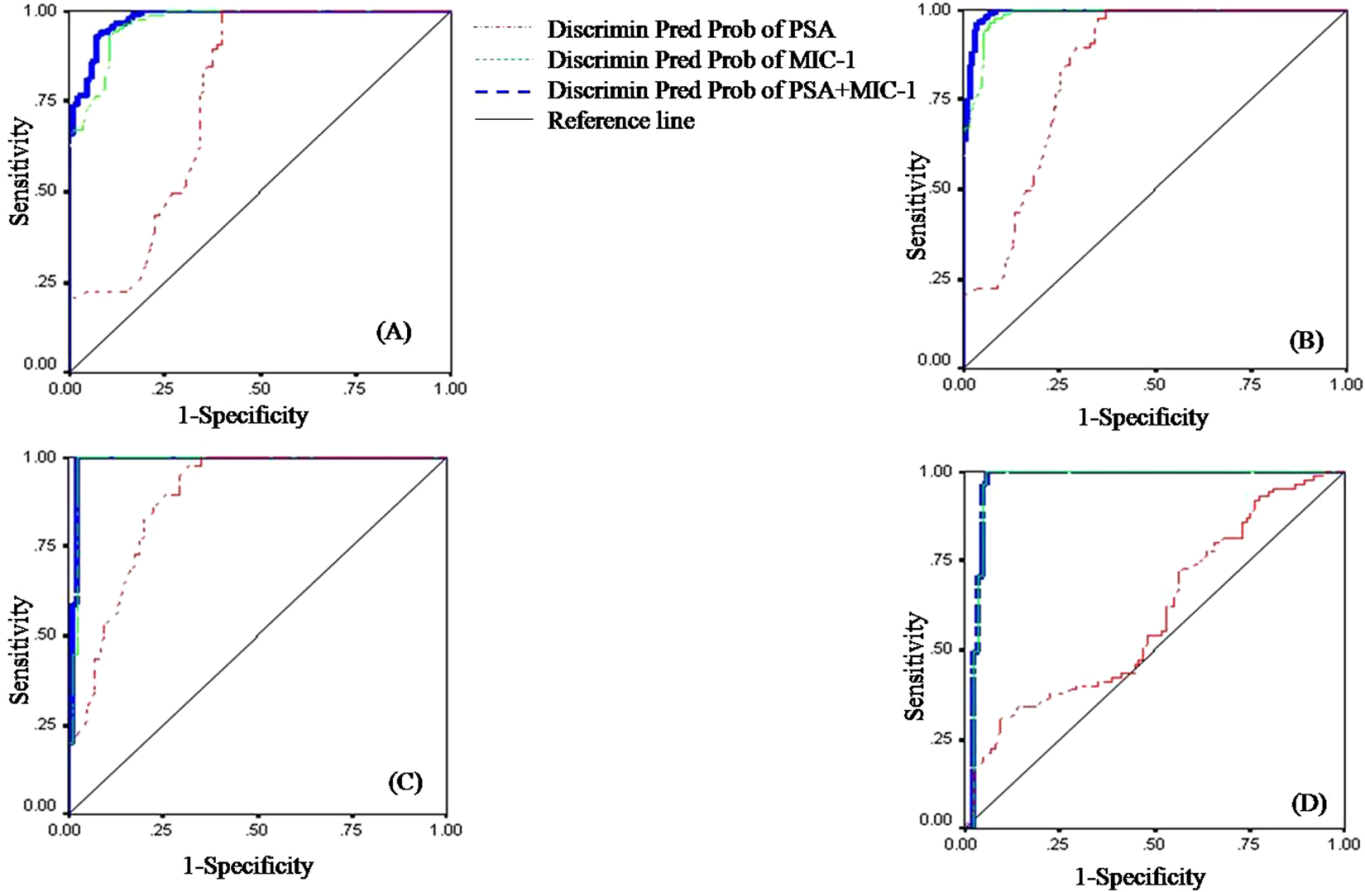
Appraisal of AUC of ROC depicting the diagnostic accuracy based on discriminant predicted probability of PSA, MIC-1, and combination of both these protein biomarkers. AUC of ROC curves of (**A**) HC vs. BPH + PC, (**B**) HC vs. BPH, (**C**) HC vs. PC, and (**D**) BPH vs. PC, details shown in Table 2. Here, HC: healthy control; BPH: benign prostate hyperplasia; PC: prostate cancer.

Comparative statistical significance and ROC analyses of signature proteins were executed to determine the clinical usefulness of MIC-1 and to evaluate their efficiency as protein biomarkers for the prediction of PC. Table 2 exhibits the sensitivity, specificity, classification precision, statistical significance and ROC analysis results of individual biomarkers with their AUC (area under the curve). PSA can precisely classify only 74.7% of BPH cases compared to HC, 62.7% of BPH + PC cases compared to HC, 71.8% of PC cases compared to HC, and 56.5% of PC cases compared to BPH. In contrast, only MIC-1 can precisely classify 91.2% of BPH cases compared to HC, 71.8% of BPH + PC cases compared to HC, 96.5% of PC cases compared to HC, and 87.6% of PC cases compared to BPH. Combinations of PSA and MIC-1 biomarkers were also executed. The outcome revealed that the combination can precisely classify 91.8% of BPH cases compared to HC, 74.5% of BPH + PC cases compared to HC, 94.7% of PC cases compared to HC, and 87.6% of PC cases compared to BPH. Sensitivity, specificity, AUC of ROC, and statistical significance are summarized in Table 2 and Fig. 4. Table 2 indicates that the individual MIC-1 measures produce better statistical outcomes than PSA. Moreover, the combination of PSA and MIC-1 measures reveal quite good differentiating biomarkers for PC with respective controls. Western blotting and ELISA based on chosen latent biomarkers and subsequent ROC analysis revealed that PC not only secretes a signature protein biomarker but also that this protein biomarker can be used to distinguish it as well.

**Table 2:** Statistical outcome of different group of classifications from respective controls.

Variables	HC vs BPH + PC				HC vs BPH				HC vs PC				BPH vs PC			
	*Sen.(%)	*Spe.(%)	ROC(Area)	*p*-value	*Sen.(%)	*Spe.(%)	ROC (Area)	*p*-value	*Sen.(%)	*Spe.(%)	ROC (Area)	*p*-value	*Sen.(%)	*Spe.(%)	ROC (Area)	*p*-value
PSA	99.9	44.2	0.836	<0.001	99.9	49.6	0.763	<0.001	99.9	43.5	0.882	<0.001	81.2	31.8	0.599	<0.001
MIC-1	99.9	57.6	0.983	<0.001	92.9	89.4	0.967	<0.001	99.9	92.9	0.984	<0.001	99.9	75.3	0.967	<0.001
PSA + MIC-1	99.9	61.8	0.991	<0.001	94.1	89.4	0.979	<0.001	99.9	89.4	0.986	<0.001	99.9	73.3	0.967	<0.001

HC, control subject; BPH, benign prostatic hyperplasia; PC, prostate cancer; *Sen., sensitivity; *Spe., specificity.

PSA based statistical outcomes of different cohorts.

MIC-1 based statistical outcomes of different cohorts.

PSA + MIC-1 based statistical outcomes of different cohorts.

## Discussion

To develop diagnostic and therapeutic interventions, it becomes obligatory to comprehend molecular changes associated with carcinogenesis, tumor growth, invasion and metastasis. One such interesting bio-molecule MIC-1 has been acknowledged to be overexpressed in epithelial cancer, breast, gastric, colorectal and prostate cancer^17,21^. MIC-1 elevated levels have been reflected in blood and allied to tumor development and its progression^18,22,23^. In this double-blind study, WB and ELISA results showed significantly increased levels of MIC-1 as an indispensable tool to discriminate PC cases from HC and BPH cases. Similar results were reported in p-Chip-based MIC-1 immunoassays, annotation- and sequence analysis-based approaches^17,19^ in known samples. Studies have also reported augmented levels of MIC-1 in colon and pancreatic ductal adenocarcinoma^23,24^; hence MIC-1 may have the potential to be used as a reliable biomarker for identification of PC and lead to significantly lower numbers of unnecessary prostate biopsies. However another study revealed reduced levels of serum MIC-1 in PC cases^18^. This was contradictory to our results and other studies. We observed diminished expression of MIC-1 protein in BPH compared to PC comparable to the findings reported by Tawfik *et al*.^25^. The findings of previous and present studies epitomize the gap and lend support in favor of increased levels of MIC-1 in PC cases as a potent biomarker to predict onset and progression of PC.

In a recent study, a p-chip-based immunoassay revealed that MIC-1 was at an elevated expression in androgen-sensitive cell lines compared to androgen-insensitive PC cell lines. Further, in the same study, expression of MIC-1 was found to be low compared to control BPH cells. These studies corroborate with a quantitative RT-PCR-based study in which high expression of MIC-1 mRNA was observed in high Gleason Score PC cases^25^.

We also found that augmented serum MIC-1 levels were an independent marker of the presence of PC and tumors with a Gleason score of 6 to 10, and exhibit an outstanding correlation with metastatic progression of tumor together with the presence of bone metastases^16,25,26^.

Although there is a sturdy relationship between MIC-1 expression and epithelial tumors, little is known about its role and the signaling pathways by which it executes its role. The functions of MIC-1 might be diverse depending upon the molecular milieu. Few studies have reported MIC-1 for an antitumorigenic role via both p53-dependent and p53- independent pathways, whereas other studies have emphasized its tumorigenic role via ERK1/2 signal pathway in androgen receptor (AR)-positive PC^25^. MIC-1 Probably weakens the cell matrix and cell-cell adhesion and stimulates partial cell detachment via RhoE and certain gene expression in PC cell lines^25^. Moreover, MIC-1’s dissimilar character resembles that of the TGF-β superfamily which acts as a tumor suppressor during the early stages of tumor development and a metastasis promoter during tumor progression^27^.

Although we found augmented levels in this double-blind study of MIC-1 in PC compared to BPH and HC, the biological role of MIC-1 is still not very clear. It may be that the different roles of MIC-1 are influenced by different factors such as the nature of tumor, tumor stage, origin tissue, interaction with tumor micro environments and several other factors. Another possibility is the presence of an assorted form of MIC-1 that may vary according to tumor type, stage and its progression and intracellular processing of MIC-1 that eventually manages the ratio of MIC-1 to remain localized in tumor microenvironments and diffused into circulation. MIC-1 might also aid in tumor dissemination via its reductive effect on cell adhesion, an important event essential for the expansion of metastatic cancer cells^21,28,29^. Macrophages may play a vital role in regulating MIC-1 levels in PC, as the secretion of tumor necrosis factor-α by activated macrophages is restrained by MIC-1 and may influence tumor-killing activity of these cells^30^. Using Toll-Like Receptor (TLR) agonists as a tool to target macrophages^31,32^ might control the physiological environment leading via the modulating MIC-1 role for tumor progression and inhibition^33^.

## Conclusions

We think that MIC-1 expression is associated with PC. In our double-blind study, we showed that MIC-1 protein expression is higher in PC cases compared to BPH and HC. MIC-1 may have potential to be used as a reliable biomarker for diagnosis of PC. Our study also re-evaluates the contradictory findings and showed increased levels of MIC-1 in PC. The molecular mechanism of MIC-1 in PC is not clear; further studies are definitely required to determine the signaling pathways of MIC-1 in PC and its biological significances in relation to PC development and carcinogenesis. Similar study with an even extra-large sample size is warranted to further validate the utility of MIC-1 as a serum-based predictor of PC before drawing any conclusion.

## Methods

### Patients and sample collection

This is a retrospective study. The study was approved by the institutional ethics committee of Centre of Biomedical Research, SGPGIMS, and King George’s Medical University, Lucknow, UP, India. The written informed consent was obtained from all the subjects before enrolment in the study. All methods were performed in accordance with the relevant guideline and regulations. We evaluated all the subjects using the following clinical laboratory variables: serum PSA level (ng/mL), abnormal Digital Rectal Examination (DRE) (I, II, and III grades), and trans-rectal ultrasound (TRUS) (hypoechoic, hyperechoic, and isoechoic lesion denoted as 1, 2, 3, respectively). Low grade (LG) PC or High grade (HG) PC was determined using histopathology-based Gleason Score (GS). To interpret the PC pathology, World Health Organization (WHO) guidelines were executed. Inclusion criteria required that subjects had not received any medication or suffered any comorbidity. Exclusion criteria were as follows: diabetes mellitus, hypertension, tuberculosis, endocrinal, renal infections, cardiovascular diseases, or other malignancies. All clinical information of participants was safely kept with the clinicians without revealing to the research team to avoid the unrecognized bias.

Subjects were kept fasting overnight, followed by collection of venous blood specimens between 7.00 AM to 8.00 AM, to minimize the influence of dietary factors. The study comprises 240 subjects: HC (n = 80), BPH (n = 75) and PC (n = 85). To facilitate clotting; collected blood samples (5.0 mL) were kept in vacutainer tube for 30 min at room temperature (RT). Afterwards samples vial were centrifuged at 3000 g, 4 °C for 15 min. The supernatant serum were separated and stored at −80 °C till further experimentation.

### Affinity Depletion of Serum Samples

Fifty µL serum samples from each subjects were diluted with commercially available Buffer A (Agilent Technologies, Wilmington, DE) at 1:5 ratio and centrifuged for 15 min at 15,000 g to remove particulate matter and lipids. Afterwards, 200 µL of the diluted sample was chromatographed through a Multiple Affinity Removal System (MARS-Hu6) Column (Agilent Technologies) at a flow rate of 0.5 ml/min. The flow through fraction embrace serum depleted the six most abundant proteins (albumin, alpha-1 antitrypsin, haptoglobin, transferring, IgA, and IgG) according to the manufacturer’s instructions and protocols. The bound proteins were eluted from the column with buffer B (Agilent Technologies). The abundant proteins depleted sera were collected and stored at −80 °C until time of analysis.

### Western Blotting

Western blotting was executed using depleted serum obtained from fifteen blinded and randomly chosen subjects. The proteins were quantized using Bradford’s assay. Equal amounts of proteins (5 µg) were loaded on 12% polyacrylamide gel and run with 4% stacking gels at the end. Electrophoresis was done using a Mini Protean 3 electrophoresis system (Bio-Rad), first for 30 min at 80 V, followed by 160 V for the period of the run and chilled to 10 °C. The proteins were subsequently transferred to nitrocellulose membrane with the help of an ice-cold transfer buffer. The blots were blocked in TBS-T containing 5% skimmed dried milk, washed thrice in TBS-T buffer, incubated overnight with primary antibody (polyclonal antibody against human MIC-1 R&D System); washed thrice in TBS-T buffer followed by incubation with anti-goat horseradish-conjugated secondary antibody (R&D Systems). The blots were analysed and visualized using chemiluminescent assay.

### Enzyme Linked Immunosorbent Assay (ELISA)

To validate the result of WB, ELISA- based experiments were executed blindly on depleted sera from the original study cohort and also from independent depleted serum samples in duplicate. The experiment was carried out on Nunc-Immuno 96 Micro-Well plates, incubated with polyclonal antibody against human MIC-1 at 4 °C overnight. Following the washing of the unbound antibody, 100 µl of serum samples from each categories (HC, BPH, and PC) were added to an ELISA plate in dilution 1:20 in 5% BSA and kept for two hours at RT. This experiment was processed for incubation with 100 µl HRP-conjugated secondary antibody to each well and incubated for 45 min at RT. Afterwards, 100 µl of 3, 3′, 5, 5′-tetramethylbenzidine (TMB) was added to each well and incubated for 30 min at RT; the reaction was accomplished adding 50 µl of stop solution to each well. The optical density was measured with absorbance at 450 nm using an iMARK Bio-Rad Micro-plate Reader.

To confiscate the earlier contrasting observations, this double-blind study was executed to protect against experimental bias of known samples. Clinical evaluations of all the cases were undisclosed to participating subjects and to research team members to avoid unrecognized bias. Only the clinicians and statistician knew about the clinical appraisals. All blinded samples were processed and executed for proteomics experiments by the research team. The research team handed over all the measures of Western blot and ELISA outcomes to the clinician and statistician to execute the proper statistical analysis.

### Statistics

Results are presented as mean ± standard deviation (SD). The three groups – HC, BPH, and PC were compared by one-way ANOVA followed by a post hoc Student−Newman−Keuls multiple comparisons test using Graph Pad InStat 3.0 to identify differentially expressed MIC-1 proteins. Precision classification and receiver operating characteristic (ROC) curve analysis were conducted for clinical utility, sensitivity, specificity, and to verify the robustness of the differentially expressed proteins for discriminating specific cohorts.

## Electronic supplementary material

suplplementary information
