# Supplementary material for: Relevance of MIC-1 in the Era of PSA as a Serum Based Predictor of Prostate Cancer: A Critical Evaluation
Source: Sci Rep. 2017 Dec 4;7:16824. doi: 10.1038/s41598-017-17207-2 (PMC5715056; doi:10.1038/s41598-017-17207-2)
Supplement: Supplementary file 1 — suplplementary information [file 41598_2017_17207_MOESM1_ESM.pdf]

Relevance of MIC-1 in the Era of PSA as a Serum Based Predictor of Prostate Cancer: A Critical Evaluation

Navneeta Bansal[1,3], Deepak Kumar[2], Ashish Gupta[2*], Deepak Chandra[3], Satya Narain Sankhwar[1], Anil Mandhani[4]

[1]Department of Urology, King George's Medical University, Lucknow, India
[2]Centre of Biomedical Research, SGPGIMS Campus, Lucknow, India
[3]Department of Biochemistry, Lucknow University, Lucknow, India
[4]Department of Urology, Sanjay Gandhi Post Graduate Institute of Medical Sciences, Lucknow, India

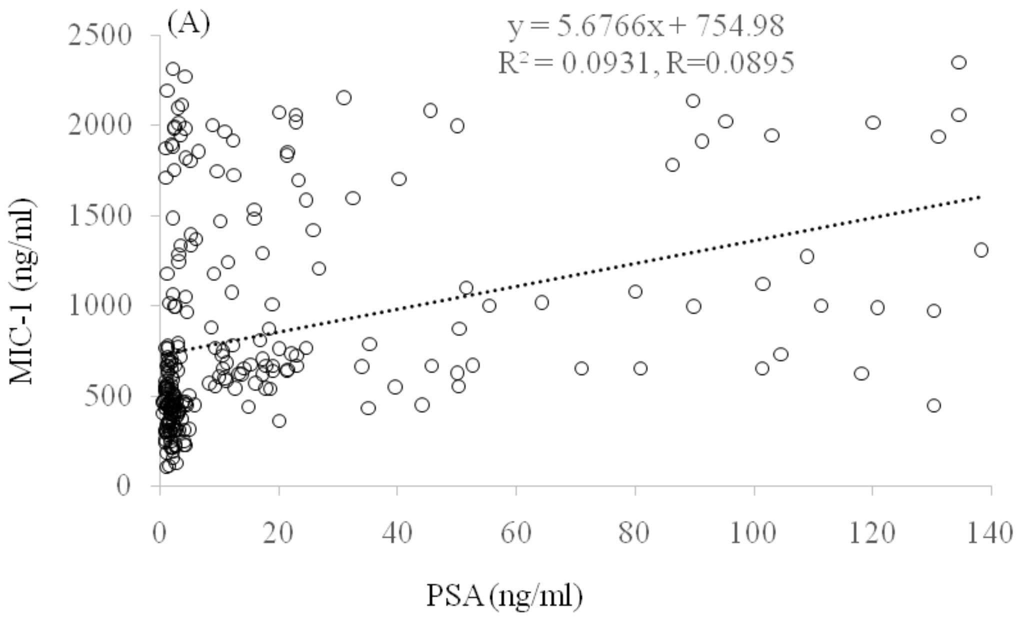

SUMMARY OUTPUT

Regression Statistics	
Multiple R	0.305183
R Square	0.093136
Adjusted R Square	0.089552
Standard Error	30.4599
Observations	255

ANOVA

	df	*SS*	*MS*	*F*	*Significance F*
Regression	1	24107.66	24107.66	25.98352	6.74845E-07
Residual	253	234734.9	927.8058		
Total	254	258842.5			

	Coefficients	*Standard Error*	*t Stat*	*P-value*	*Lower 95%*	*Upper 95%*	*Lower 95.0%*	*Upper 95.0%*
Intercept	5.062142	3.372837	1.500856	0.13464	-1.580272317	11.70456	-1.58027	11.70456
X Variable 1	0.016407	0.003219	5.097403	6.75E-07	0.010068215	0.022746	0.010068	0.022746

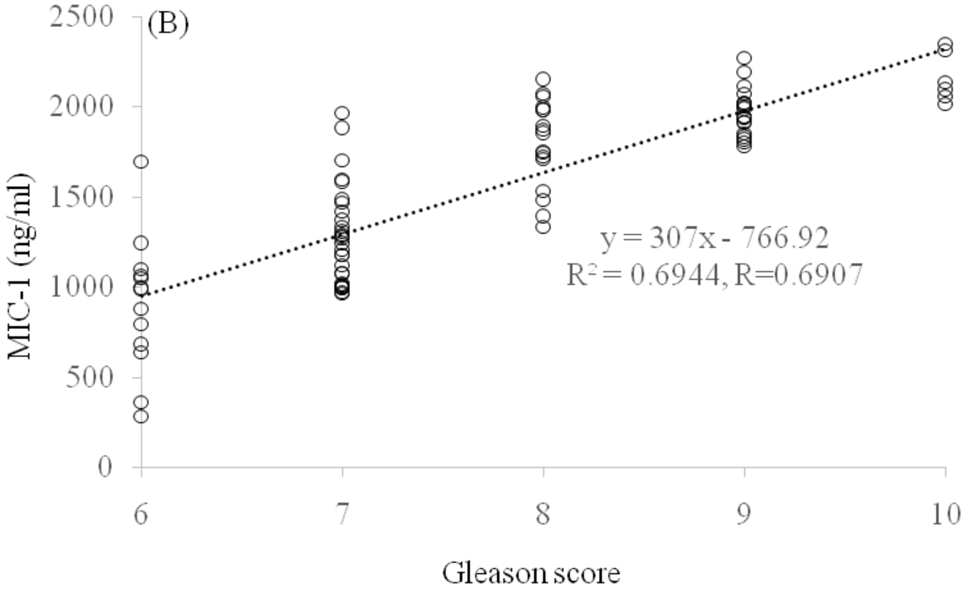
(B)
2500
2000
1500
1000
500
0
MIC-1 (ng/ml)
6
7
8
9
10
Gleason score
y = 307x - 766.92
R² = 0.6944, R=0.6907

SUMMARY OUTPUT

Regression Statistics	
Multiple R	0.833311
R Square	0.694408
Adjusted R Square	0.690726
Standard Error	0.658147
Observations	85

ANOVA

	df	*SS*	*MS*	*F*	*Significance F*
Regression	1	81.69504	81.69504	188.6038	4.48196E-23
Residual	83	35.95202	0.433157		
Total	84	117.6471			

	Coefficients	*Standard Error*	*t Stat*	*P-value*	*Lower 95%*	*Upper 95%*	*Lower 95.0%*	*Upper 95.0%*
Intercept	4.089549	0.27283	14.98935	2.47E-25	3.54690018	4.632197	3.5469	4.632197
X Variable 1	0.002262	0.000165	13.73331	4.48E-23	0.001934306	0.002589	0.001934	0.002589
